# A Case Report of Tracheobronchomalacia Requiring Stenting and Extracorporeal Membrane Oxygenation Treatment

**DOI:** 10.1155/crcc/6232860

**Published:** 2026-08-19

**Authors:** Hiroyuki Tanaka

**Affiliations:** ^1^ Department of Emergency and Critical Care Medicine, National Hospital Organization Kyoto Medical Center, Kyoto, Japan, hosp.go.jp

**Keywords:** extracorporeal cardiopulmonary support (ECMO), stenting, tracheobronchomalacia (TBM)

## Abstract

**Background:**

Tracheobronchomalacia (TBM) is characterized by the excessive expiratory collapse of the central airway due to structural weakness or membranous redundancy of the tracheal and bronchial cartilages. TBM may present with anatomical and physiological compromise of the airways. Aggressive treatments for severe respiratory failure include mechanical ventilation and extracorporeal membrane oxygenation (ECMO). Interventional approaches include short‐term silicone stent trials to predict surgical benefits and definitive procedures, such as tracheobronchoplasty.

**Case Presentation:**

An Asian male patient in his 60s with a history of COPD receiving home oxygen therapy (HOT) was transferred to our hospital for dyspnea. His baseline symptoms were classified as Hugh–Jones Class III. Upon admission, the partial pressure of oxygen in arterial blood/fraction of inspired oxygen (PaO_2_/FiO_2_) ratio was 110. Computed tomography (CT) revealed bilateral narrowing of the trachea and bronchi. Estimated expiratory airway collapse for repeated CT scans was 75%, and limited dynamic 3D airway reconstruction demonstrated expiratory tracheal collapse, supporting the diagnosis of TBM. The patient received high‐flow nasal oxygen therapy but gradually deteriorated and required mechanical ventilation. Biopsy revealed expanded submucosal fibrosis. The patient′s respiratory status deteriorated on the 11th day after admission with no improvement in any ventilator settings. Venous–venous ECMO was performed. Airway stenting was performed on the 20th day of admission. ECMO was discontinued on the 28th day, and the patient was weaned from the ventilator on the following day (Day 29).

**Conclusions:**

This case illustrates that in severe TBM, where both airway patency and gas exchange are compromised, ECMO may serve as a strategic bridge to definitive airway intervention. This case illustrates features of both an anatomically difficult airway (critical central airway collapse) and a physiologically difficult airway (severe hypoxemic respiratory failure), highlighting the importance of managing both airway anatomy and physiology simultaneously. Early extracorporeal support may be considered in carefully selected patients with reversible causes of severe respiratory failure.

## 1. Background

Tracheobronchomalacia (TBM) is characterized by structural weaknesses that lead to excessive collapse of the trachea and bronchi during exhalation [1], resulting in a marked reduction in airway diameter during expiration. Nonspecific respiratory symptoms, such as dyspnea, cough, wheezing, stridor, and impaired secretion clearance, may be present in patients with TBM. Its etiology can be congenital or acquired. COPD, asthma, collagen vascular diseases such as relapsing polychondritis, obesity‐related anatomical changes, severe recurrent infections, aspiration events linked to gastroesophageal reflux disease (GERD), and prolonged airway manipulation have all been implicated in the pathogenesis of acquired TBM, with most studies pointing to a reversible clinical course [2, 3]. Diagnoses include clinical assessments such as pulmonary function tests, radiologic imaging, and endoscopic evaluation. Treatment for respiratory failure includes mechanical ventilation and ECMO in severe cases, and interventional and surgical management, such as airway stenting and tracheobronchoplasty, may be indicated in selected cases. We encountered a unique case of TBM that required airway stenting and ECMO with favorable outcomes.

## 2. Case Presentation

An Asian male patient in his 60s with a history of COPD receiving home oxygen therapy (HOT) was transferred to our hospital for dyspnea. He had a history of smoking two packs of cigarettes per day. On admission, the patient had a respiratory rate of 34 bpm and a pulse oximetry reading of 88% under 12 L/min oxygen administration. His blood pressure was 120/78 mmHg, his heart rate was 109 bpm, and he was afebrile, with a body temperature of 36.9°C (103.5°F). Auscultation revealed slight bilateral wheezing in all lung fields. Blood tests revealed elevated white blood cell counts and C‐reactive protein levels. Arterial blood gas analysis showed Type I respiratory failure with a partial pressure of oxygen in arterial blood/fraction of inspired oxygen (PaO_2_/FiO_2_) ratio of 180 and a PaCO_2_ of 40 mmHg. Radiographic imaging revealed no infiltrates or atelectases. Computed tomography (CT) revealed bilateral narrowing of the trachea and bronchi. Complete inspiratory/expiratory CT could not be obtained because of respiratory instability, but estimated expiratory airway collapse for repeated CT scans was 75%, and limited dynamic 3D airway reconstruction demonstrated expiratory tracheal collapse, both supporting the diagnosis of TBM.

**Figure 1:**
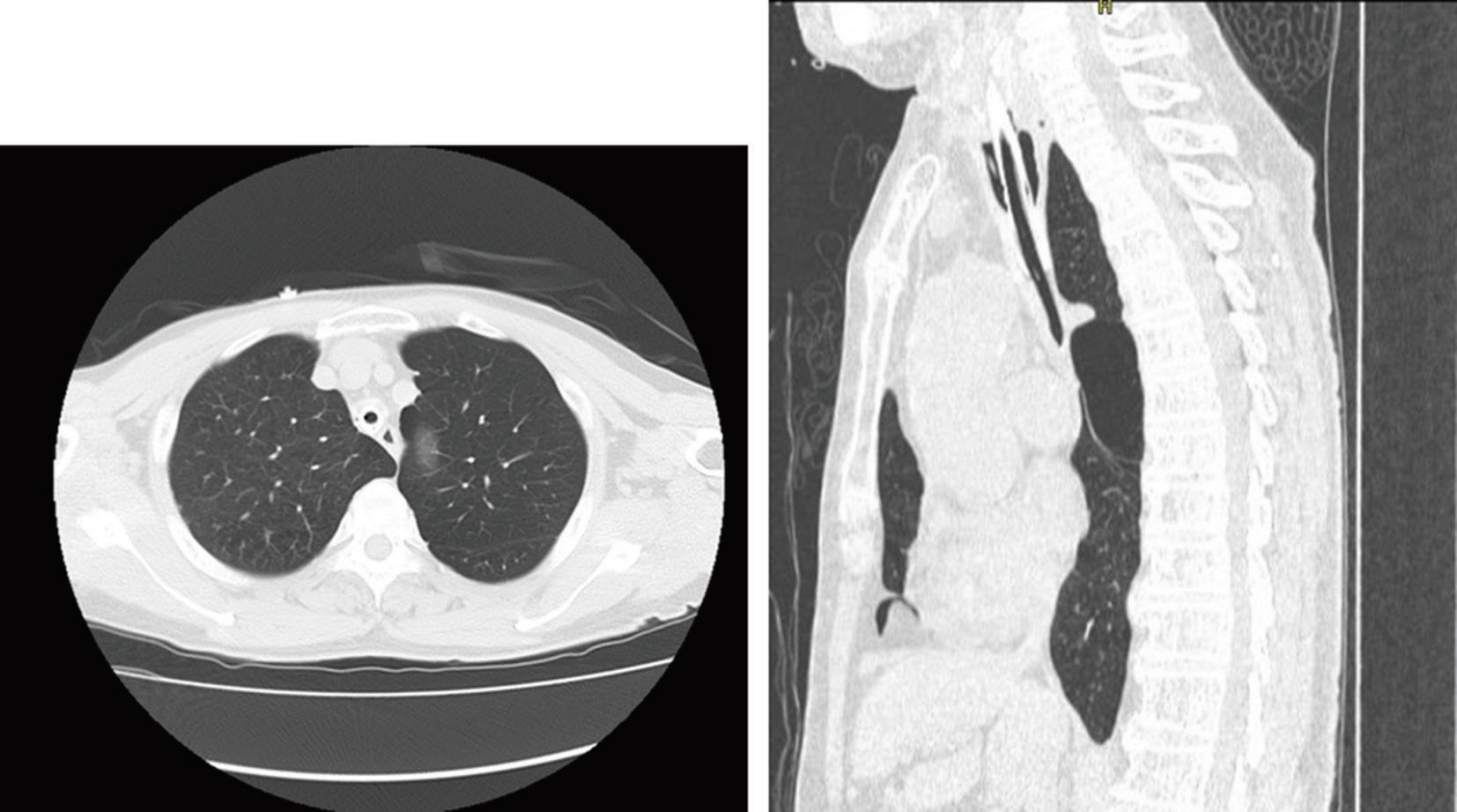
Imaging studies performed after intubation. CT scan shows narrowing of the lumen, just adjacent to the endotracheal tube.

The patient initially received high‐flow nasal oxygen therapy; however, his respiratory condition deteriorated rapidly, with a PaO_2_/FiO_2_ ratio of 110 on the 2nd admission day. The patient was intubated and received mechanical ventilation on the same day. A repeated CT scan shows narrowing of the lumen, just adjacent to the endotracheal tube (Figure 1). A biopsy was performed to rule out other underlying diseases, such as malignancy.

The ventilator was set to pressure control assist‐control mode with a positive end‐expiratory pressure (PEEP) of 10–12 cmH_2_O, pressure support of 6–10 cmH_2_O, a respiratory rate of 20/min, an I:E ratio of 1:2, and an FiO_2_ of 0.35–0.50. High PEEP was necessary for airway patency, but expiratory flow restrictions made it difficult for adequate ventilation and recruitment. The PaO_2_/FiO_2_ ratio remained relatively unchanged, ranging from 150 to 250 until the 11th day of admission.

**Figure 2:**
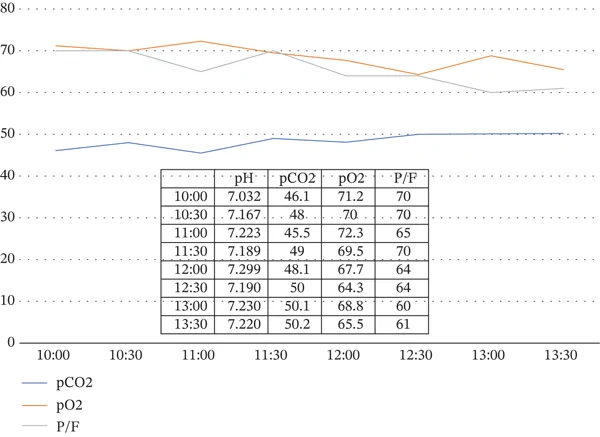
Respiratory trend before initiation of ECMO. Trends in the pH, PaO_2_, PaCO_2_, and FiO_2_ ratio are shown. The inlying table shows actual figures.

**Figure 3:**
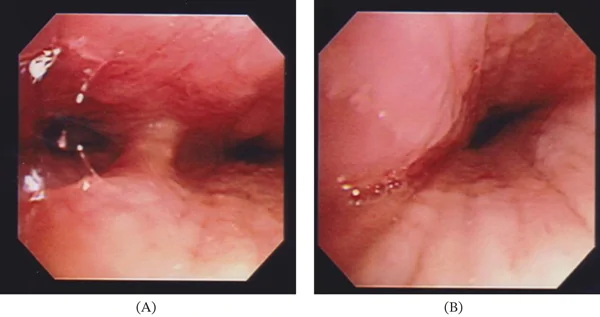
Bronchofiberscopy images, before stenting. (A) First bifurcation and (B) right upper lobe (S2). Notice the narrowing of the lumens.

On the 11th day of admission, the patient′s respiratory condition deteriorated suddenly; adequate tidal volume could not be achieved despite high ventilator settings, with a maximum PEEP of 15 cmH_2_O and a maximum pressure above PEEP of 20 cmH_2_O. PaO_2_/FiO_2_ ratio was reduced to 60. In addition, respiratory acidosis due to PaCO_2_ retention became evident as shown in Figure 2. CT and bronchoscopy revealed further bilateral narrowing of the trachea and bronchi extending to the segmental bronchi, as shown in Figure 3. Mechanical ventilator settings did not improve the patient′s respiratory condition, and venous–venous ECMO therapy was introduced. The PaO_2_, PaCO_2_, and PaO_2_/FiO_2_ ratios are shown in Figure 2.

The ECMO flow was set to 3.5 L/min, with a sweep gas flow of 3 L/min and an FiO_2_ setting of 0.8. Histopathological examination of the biopsy specimen revealed expanded submucosal fibrosis with no evidence of malignancy. Combined with the sudden deterioration in the PaO_2_/FiO_2_ ratio with no other evident diseases, a clinical diagnosis of TBM was made. Systemic steroids were administered with no other definitive treatment or clinical improvement.

Definitive treatment was discussed among the care team. Tracheobronchoplasty was not considered feasible during the acute phase because of severe respiratory failure and hemodynamic instability.

**Figure 4:**
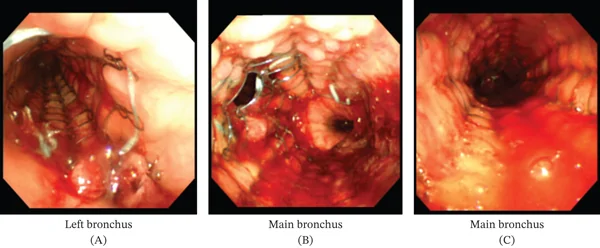
Bronchofiberscopy images, poststenting. (A) Left bronchus, (B) main bronchus—first bifurcation, and (C) main bronchus.

**Figure 5:**
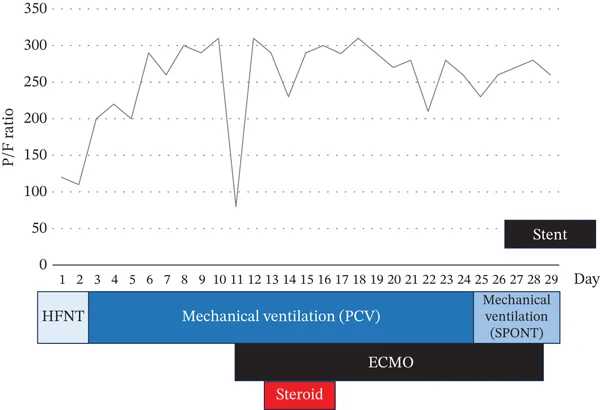
Clinical course of the patient. Trends in the PaO_2_/FiO_2_ ratio and ECMO flow are shown.

Airway stenting was performed on the 20th admission day. As TBM is considered a reversible pathology, a tracheobronchial uncovered stent was selected: a 14 mm, length 40 mm stent for the left bronchus, from the first bifurcation to the secondary bifurcation, and a 20 mm, length 60 mm stent for the main trachea, proximal to the first bifurcation. The poststenting fiberscopic findings are shown in Figure 4. After stenting, tidal volume showed an average improvement of 48%, airway resistance decreased by an average of 53%, and lung compliance improved by an average of 35%. ECMO was successfully discontinued on the 8th day (28th admission day). The patient was free of ventilator support on the 29th day, was transferred to the general ward on the 43rd day, and was discharged on the 45th day. The patient′s clinical course is shown in Figure 5. The patient was followed up by the respiratory department every 3 months after discharge for 2 years, but no relapse of symptoms was documented.

## 3. Discussion

Respiratory failure in TBM has many causes, with dynamic airway collapse and expiratory flow limitations being the major factors [1]. PEEP may be an effective treatment for distal TBM but not for central TBM [1, 2]. Additionally, continuous PEEP may result in hemodynamic instability and compromise ventilation. Combination therapy for TBM has been discussed in many studies, but this is a unique report demonstrating successful combined airway stenting and ECMO therapy for a patient presenting with consequent Type 2 respiratory failure due to TBM per se and a favorable prognosis at discharge. The treatment strategies and duration for TBM vary according to the literature, with some durations as long as 64 days [4]. Furthermore, very few case reports describe ECMO use for TBM, most of which involve ECMO for stent‐related complications and treatment [5, 6]. ECMO may also be indicated for severe respiratory failure due to airway collapse and inadequate ventilation in severe TBM cases, provided that the cause is considered irreversible. Medications for TBM have been insufficiently studied, although some reports suggest that steroids reduce inflammation [1]. Airway stenting is performed under endoscopic guidance. Various stents are available, including covered and noncovered endotracheal stents. However, evidence supporting the superiority of one stent over another is scarce. Covered stents are frequently used to treat tracheal stenosis in terms of flexibility and rigidity. In contrast, short‐term airway stabilization with silicone stents has demonstrated measurable improvements in several patient‐reported domains in severe TBM cases. These benefits were observed even in patients with significant comorbid conditions, such as COPD, indicating that coexisting pulmonary disease is not an absolute barrier to achieving subjective improvement during the intervention period [7]. In this case, considering the expected treatment duration and institutional restrictions, an uncovered stent was used. ECMO is an invasive treatment for respiratory failure that is unresponsive to conventional treatments. Several guidelines have been established for its indication [8]. Hypoxemic respiratory failure (PaO_2_/FiO_2_ < 80 mmHg after optimal medical management or hypercapnic respiratory failure) is indicated. In our case, the former was applicable where positive pressure ventilation was ineffective, and airway collapse prevented tidal ventilation. In addition, respiratory acidosis due to PaCO_2_ retention became evident, as shown in Figure 2, which is another rationale for ECMO. Reversibility is the primary decision factor. Contraindications included severe underlying diseases. In our case, the concern for ECMO introduction was COPD; however, ECMO introduction was decided based on the reversibility of the TBM. ECMO provides physiological support while definitive airway management is undertaken. No clear guidelines exist regarding the timing of ECMO initiation; however, a longer treatment duration before ECMO suggests a less favorable outcome. In this case, the patient underwent ECMO therapy for < 24 h after respiratory deterioration. This decision was made by the care team. The duration of ECMO is primarily determined by the cause of respiratory failure, with one report describing ECMO support for 64 days [9]. Quality of life outcomes in patients with TBM are closely tied to both the symptomatic burden at baseline and the effectiveness of therapeutic interventions. Device‐related complications can directly influence quality of life. Partial stent obstruction, documented in 21 cases, can cause the recurrence of airflow limitation symptoms until addressed [7]. Stent migration, as reported in 10 instances, may also unpredictably alter airflow dynamics and expose patients to renewed obstruction or cough exacerbation. Interventional pulmonology and thoracic surgery collaboration are crucial in managing TBM. In our case, follow‐up was performed for 2 years, with no recurrence. The patient had been receiving HOT under the same conditions before admission (2 L/min), and symptoms remained largely unchanged, with Hugh–Jones Class III–IV functional status. There are several limitations of this report. Firstly, due to the patient′s respiratory instability, complete inspiratory/expiratory CT could not be obtained for the definite diagnosis of TBM. Secondly, this was a single case report, which may require further case accumulation for concrete conclusions.

## 4. Conclusion

Though this was a single case report, it illustrates that in severe TBM, where both airway patency and gas exchange are compromised, ECMO may serve as a strategic bridge to definitive airway intervention. Both an anatomically difficult airway (critical central airway collapse) and a physiologically difficult airway (severe hypoxemic respiratory failure) highlight the importance of managing both airway anatomy and physiology simultaneously. Early extracorporeal support may be considered in carefully selected patients with reversible causes of severe respiratory failure.

## Author Contributions

H.T. was the major contributor to writing the manuscript.

## Funding

No funding was received for this manuscript.

## Consent

Written informed consent was obtained from the patient for the publication of this case report and the accompanying images.

## Conflicts of Interest

The author declares no conflicts of interest.

## Data Availability

Data sharing is not applicable to this article as no datasets were generated or analyzed during the current study.
